# Adjuvant Botulinum Toxin Type A on the Management of Giant Hiatal Hernia: A Case Report

**DOI:** 10.7759/cureus.53836

**Published:** 2024-02-08

**Authors:** Catarina D Henriques, Egon F Rodrigues, Lucia Carvalho, Ana Marta Pereira, Mário Nora

**Affiliations:** 1 Department of General Surgery, Centro Hospitalar de Entre o Douro e Vouga, Santa Maria da Feira, PRT

**Keywords:** giant hiatal hernia, intra-abdominal pressure, adjuvant therapy, hiatal hernia with loss of domain, botulinum toxin type a, paraesophageal hiatal hernia, hiatal hernia repair

## Abstract

The management of giant hiatal hernias (HHs) remains challenging and is associated with a high risk of recurrence. Currently, several strategies are used to reduce recurrence, and a newly proposed trend is the administration of adjuvant botulinum toxin type A (BTX), a procedure already performed in complex ventral hernias. Here, we present a case of a 63-year-old man with a giant paraesophageal HH type IV containing the entire stomach and transverse colon with loss of domain, who underwent adjuvant BTX and subsequently laparoscopic hiatoplasty with a biological mesh with partial fundoplication. At six months’ follow-up, the patient reported a significant improvement in the quality of life without dysphagia or gastroesophageal reflux and with a good respiratory function. A control computed tomography was performed, which documented a partial recurrence of HH, completely asymptomatic. This clinical case showed the successful treatment of a giant HH using adjuvant BTX injection to increase abdominal wall compliance as had already been described in the treatment of complex ventral hernia. Thus, the use of BTX is a promising strategy for selected cases of giant HHs mainly if there is a loss of domain; however, more case series and controlled trials are needed to show the reproducibility of the benefit of this strategy.

## Introduction

There is no consensus about the definition of a giant hiatal hernia (HH). Some authors define a giant HH when more than 30% of the stomach is herniated into the chest, while others if herniation is more than 50%. Type III and IV paraesophageal hernias are also usually considered giant HHs.

The prevalence of HHs varies between 15% and 20%, and a giant HH represents from 0.3% to 15% of all HHs. However, more precise estimates of the incidence or prevalence of giant HHs are difficult, as a consensus on the definition of giant HHs is lacking [1].

Any patient with a giant HH should be considered a surgical candidate unless comorbidities are prohibitive as a truly asymptomatic giant hernia is virtually non-existent [1,2]. However, the management of giant paraesophageal hernias remains challenging and is associated with a high risk of recurrence. Currently, several strategies are used to reduce recurrence, namely, an association of an anti-reflux procedure, excision of the hernial sac, esophageal lengthening maneuvers, interrupted non-absorbable suture with minimal tension, and use of mesh in selected cases [3].

A new strategy proposed is the administration of adjuvant botulinum toxin type A (BTX), a procedure already performed in complex ventral hernias [4]. BTX is a neurotoxin produced by *Clostridium botulinum*, and serotype A is the most powerful. This neurotoxin blocks the release of acetylcholine in the cholinergic nerve terminal, which leads to temporary muscle paralysis with muscle stretching [5]. Therefore, a decrease in intra-abdominal pressure is achieved, preventing the recurrence of ventral hernias and possibly HHs [6].

We present a case of a 63-year-old man with a giant HH who underwent adjuvant BTX and subsequently laparoscopic hiatoplasty with biological mesh with partial fundoplication.

## Case presentation

**Figure 1:**
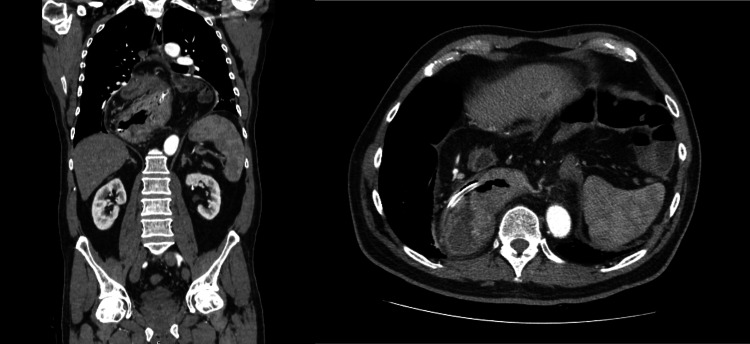
Computed tomography (coronal and transverse section) performed in the emergency department Giant HH with the pylorus positioned in a plane superior to the gastric fundus. Axial mesenteric gastric volvulus cannot be excluded.

A 63-year-old man with a giant HH diagnosed 30 years ago was referred to a general surgery consultation after an episode of upper gastrointestinal obstruction with suspected gastric volvulus. In this episode, the patient went to the emergency department due to incoercible vomiting, and the computed tomography (CT) raised the possibility of gastric volvulus (Figure 1). Exploratory laparoscopy was proposed, but the patient refused. As the patient was hemodynamically stable and had no signs of gastric ischemia on CT, he was admitted under conservative treatment. Management consisted of nasogastric intubation, intravenous fluid administration, and observation, with clinical resolution.

**Figure 2:**
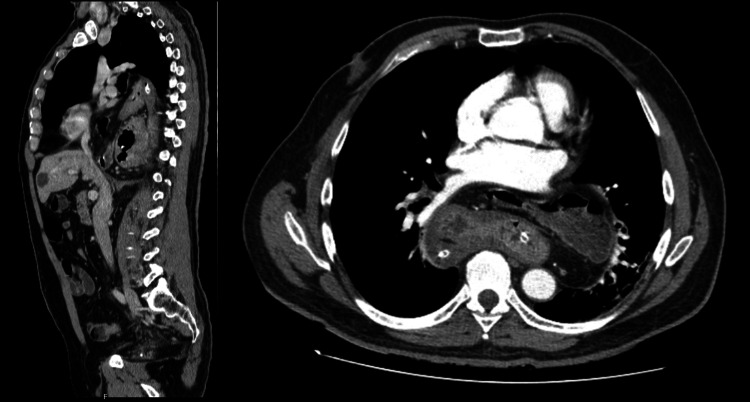
Computed tomography (sagittal and transverse section) performed to document a giant HH and to plan a treatment approach Giant HH with herniation of the fundus and gastric body (maximum intrathoracic transverse diameter of 165 mm), without evidence of volvulus, parietal thickening or signs of ischemia, and transverse colon; the neck of the hernia has a maximum transverse diameter of 43 mm.

During the consultation, the patient reported complaints of gastroesophageal reflux (GER) and occasional vomiting mainly after copious meals, about twice a week, without permanent food intolerance. The patient had a prior history of hypertension and tobacco use (10 cigarettes per day). Physical examination was unremarkable. BMI was 26 kg/m^2^. CT scan (Figure 2) and endoscopy were performed, which identified a giant paraesophageal HH type IV containing the entire stomach and transverse colon.

Preoperative preparation was focused on physical prehabilitation, kinesiotherapy, and smoking cessation. Due to the loss of domain of the HH, adjuvant therapy was undertaken with the injection of BTX to increase abdominal capacitance and reduce the risk of recurrence.

**Figure 3:**
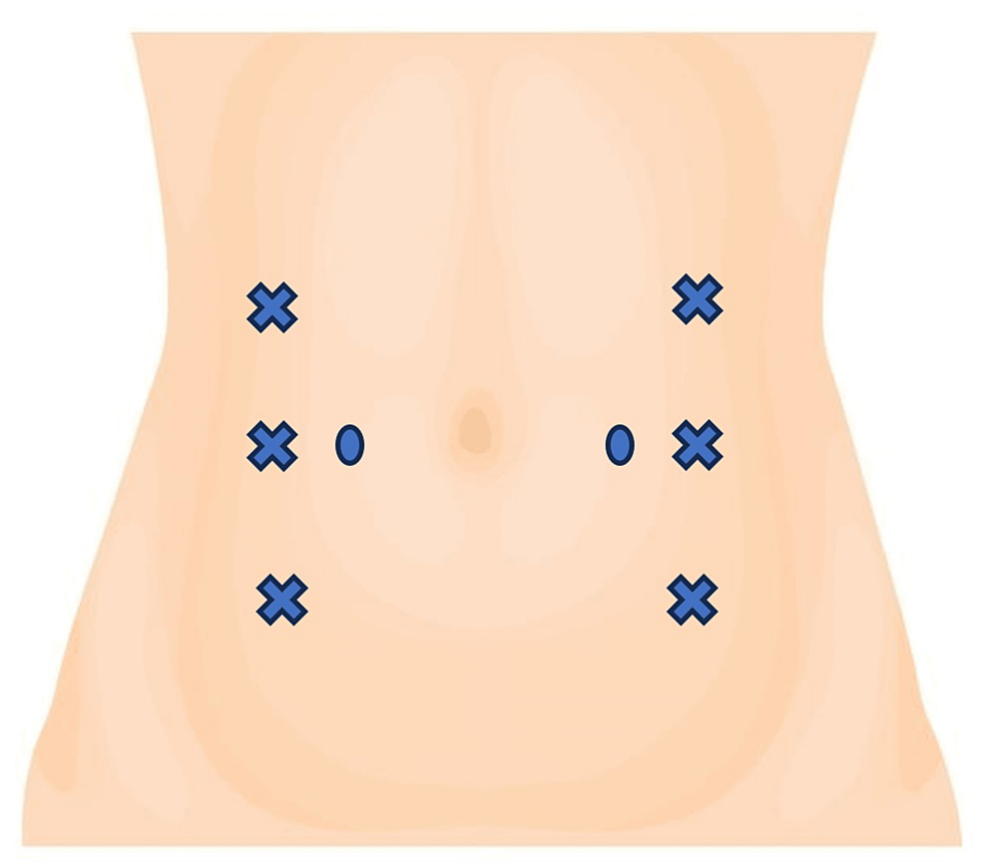
BTX injection sites Marked with cross: three points on each side in the external oblique, internal oblique, and transverse abdominal muscles; marked with circles: two points on each side retrorectum abdominal. BTX: botulinum toxin type A

Based on what has already been described for the correction of complex ventral hernias, an ultrasound-guided injection of 500 units of BTX was administered in eight separate injection sites (Figure 3): in three points on each side in the external oblique, internal oblique, and transverse abdominal muscles. Two points were added on each side of the retrorectus abdominal to promote a circumferential distention of the abdominal wall. 

**Figure 4:**
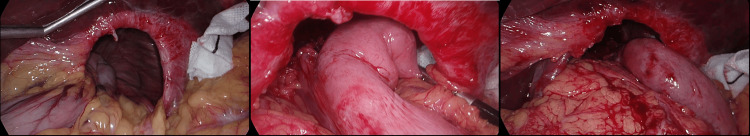
Laparoscopic hiatoplasty with biological mesh and partial fundoplication Intraoperative reduction of a giant HH with the stomach and colon in a mediastinal position.

Four weeks later, a laparoscopic hiatoplasty with biological mesh and partial fundoplication was performed. Intraoperatively, a giant HH with the stomach and colon located in a mediastinal position was confirmed (Figure 4). The crus of the diaphragm was approximated without tension with five interrupted non-absorbable stitches. Given the size of the HH, it was decided to place a "V"-shaped biological mesh fixed with a non-absorbable suture. As an anti-reflux procedure, a "short floppy Nissen" fundoplication was performed. A transhiatal drain was left to reduce the risk of seroma. There were no postoperative complications.

An oral liquid diet was initiated on the first postoperative day, the drain was removed on the third day, and the patient was discharged on the fourth day.

**Figure 5:**
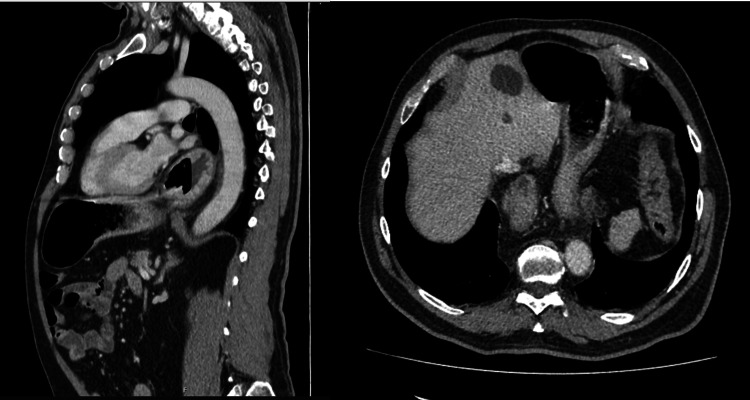
Computed tomography (sagittal and transverse section) performed during the follow-up period A partial recurrence of HH was documented.

At six months’ follow-up, the patient reported a significant improvement in quality of life without dysphagia or GER complaints and with good respiratory function. A control CT was performed (Figure 5), which documented a partial recurrence of HH. Since the patient is completely asymptomatic, a conservative attitude has been adopted. At 12 months' follow-up, the patient remained without complaints.

## Discussion

The treatment of giant HHs remains challenging, mainly due to their technical complexity and the risk of recurrence. This clinical case showed the successful treatment of a giant HH using adjuvant BTX injection to increase the abdominal compartment as already described in the treatment of complex ventral hernias of the abdominal wall [6,7]. Nonetheless, its application in the treatment of HHs was only described once [4].

Injection of BTX leads to a loss of muscle tone with subsequent muscle elongation allowing the increase of intra-abdominal volume. Therefore, a reduction of abdominal pressure is observed, which may also improve ventilation compliance [5]. In the case of giant HHs with loss of domain, it becomes even more important to reduce intra-abdominal pressure and reduce transdiaphragmatic pressure variations to reduce the risk of recurrence.

A great advantage of BTX is its safe use, with rare side effects described and usually mild, such as hematoma, pain, allergic response, rash, or injection failure [5]. Muscle paralysis reaches its maximum effect at four weeks and lasts six to nine months. Thus, the BTX administration should occur at least four weeks before surgery, as described in this case.

Furthermore, optimization of other factors that increase the risk of recurrence, such as smoking cessation and weight loss, is crucial for a good surgical result.

Another important point is the high recurrence rate of giant HHs and how this recurrence is measured. Recurrence is difficult to estimate since a recurrence in an imaging study does not necessarily correspond to a clinical recurrence. It has been described that imaging recurrence can reach 30% while clinical recurrence requiring surgical reintervention is less than 5% [3]. The only indication for performing imaging studies to document recurrence is when the patient is symptomatic [8]. However, in this case, a CT scan was requested for documentation although the patient was asymptomatic, so an expectant attitude was maintained.

A point of discussion in this case was the surgical mesh used. Hiatoplasty was performed using a biological mesh, which is usually associated with a lower risk of infection and local complications but higher recurrence rates [9]. Despite the fact that currently there is no consensus, biosynthetic meshes are gaining popularity and could be a possibility in this case, if available [10].

## Conclusions

The use of BTX is a promising strategy for selected cases of giant HHs, mainly if there is a loss of domain. Promoting a reduction of intra-abdominal pressure with an increase of intra-abdominal volume, this strategy leads to a lower risk of recurrence with a high safety profile. However, more case series and controlled trials are needed to show the reproducibility of the benefit of using adjuvant BTX in the treatment of giant HHs.
